# Targeting malaria parasites with novel derivatives of azithromycin

**DOI:** 10.3389/fcimb.2022.1063407

**Published:** 2022-11-30

**Authors:** Amy L. Burns, Brad E. Sleebs, Maria Gancheva, Kimberley T. McLean, Ghizal Siddiqui, Henrietta Venter, James G. Beeson, Ryan O’Handley, Darren J. Creek, Shutao Ma, Sonja Frölich, Christopher D. Goodman, Geoffrey I. McFadden, Danny W. Wilson

**Affiliations:** ^1^ Research Centre for Infectious Diseases, School of Biological Sciences, the University of Adelaide, Adelaide, SA, Australia; ^2^ School of Science and Technology, the University of New England, Armidale, NSW, Australia; ^3^ ACRF Chemical Biology Division, Walter and Eliza Hall Institute of Medical Research, Parkville, VIC, Australia; ^4^ Department of Medical Biology, University of Melbourne, Parkville, VIC, Australia; ^5^ Drug Delivery Disposition and Dynamics, Monash University, Parkville, VIC, Australia; ^6^ Health and Biomedical Innovation, Clinical and Health Sciences, University of South Australia, Adelaide, SA, Australia; ^7^ Healthy Mothers, Healthy Babies Program, Burnet Institute, Melbourne, VIC, Australia; ^8^ Department of Medicine, University of Melbourne, Parkville, VIC, Australia; ^9^ Central Clinical School, Monash University, Melbourne, Vic, Australia; ^10^ Department of Microbiology, Monash University, Melbourne, Vic, Australia; ^11^ School of Animal and Veterinary Science, University of Adelaide, Adelaide, SA, Australia; ^12^ Australian Centre for Antimicrobial Resistance Ecology, The University of Adelaide, Adelaide, SA, Australia; ^13^ Department of Medicinal Chemistry, Key Laboratory of Chemical Biology, Ministry of Education, School of Pharmaceutical Sciences, Shandong University, Jinan, China; ^14^ School of BioSciences, University of Melbourne, Parkville, VIC, Australia

**Keywords:** malaria, antimalarial, azithromycin, quick-killing, *Plasmodium*

## Abstract

**Introduction:**

The spread of artemisinin resistant *Plasmodium falciparum* parasites is of global concern and highlights the need to identify new antimalarials for future treatments. Azithromycin, a macrolide antibiotic used clinically against malaria, kills parasites *via* two mechanisms: ‘delayed death’ by inhibiting the bacterium-like ribosomes of the apicoplast, and ‘quick-killing’ that kills rapidly across the entire blood stage development.

**Methods:**

Here, 22 azithromycin analogues were explored for delayed death and quick-killing activities against *P. falciparum* (the most virulent human malaria) and *P. knowlesi* (a monkey parasite that frequently infects humans).

**Results:**

Seventeen analogues showed improved quick-killing against both *Plasmodium* species, with up to 38 to 20-fold higher potency over azithromycin after less than 48 or 28 hours of treatment for *P. falciparum* and *P. knowlesi*, respectively. Quick-killing analogues maintained activity throughout the blood stage lifecycle, including ring stages of *P. falciparum* parasites (<12 hrs treatment) and were >5-fold more selective against *P. falciparum* than human cells. Isopentenyl pyrophosphate supplemented parasites that lacked an apicoplast were equally sensitive to quick-killing analogues, confirming that the quick killing activity of these drugs was not directed at the apicoplast. Further, activity against the related apicoplast containing parasite *Toxoplasma gondii* and the gram-positive bacterium *Streptococcus pneumoniae* did not show improvement over azithromycin, highlighting the specific improvement in antimalarial quick-killing activity. Metabolomic profiling of parasites subjected to the most potent compound showed a build-up of non-haemoglobin derived peptides that was similar to chloroquine, while also exhibiting accumulation of haemoglobin-derived peptides that was absent for chloroquine treatment.

**Discussion:**

The azithromycin analogues characterised in this study expand the structural diversity over previously reported quick-killing compounds and provide new starting points to develop azithromycin analogues with quick-killing antimalarial activity.

## Introduction

Malaria is a mosquito-borne parasitic infection that caused ~240 million clinical cases and >600,000 deaths in 2020 (WHO, 2021). The majority of deaths occurred in children under 5 years of age in sub-Saharan Africa and were the result of *Plasmodium falciparum* infection, the most virulent human malaria parasite (WHO, 2021). A further five malaria species (Singh et al., 2004; WHO, 2021) that regularly infect humans are also major contributors to the global burden of malaria, thus future control strategies need to be effective against these species.

Over the last two decades, global distribution of insecticide treated bed nets (ITNs) and artemisinin-based combinational therapies (ACTs) have contributed to a >50% decrease in global malaria mortality (WHO, 2021). However, *P. falciparum* parasites resistant to frontline artemisinin combination therapy have emerged in Southeast-Asia and other endemic regions, including Africa, Eastern-India, South America and Papua New Guinea, increasing the chance that rates of malaria morbidity and mortality will rise (Ashley et al., 2014; Miotto et al., 2020; Balikagala et al., 2021). The spread of resistance to our most effective class of antimalarials highlights the need to identify new chemotypes with novel mechanisms of action for use in future combination therapies (Burrows et al., 2017).

The clinically used macrolide antibiotic, azithromycin, has been investigated for use as a malaria prophylactic (Andersen et al., 1998; Taylor et al., 1999) and as a potential partner drug in ACTs and intermittent preventative treatment regimens (Krudsood et al., 2000; Sykes et al., 2009; Luntamo et al., 2010; Unger et al., 2015; Chandramohan et al., 2019; Phiri et al., 2021). Azithromycin inhibits protein translation in the parasite apicoplast, a relic plastid organelle (McFadden et al., 1996), *via* binding to the peptide exit tunnel of the 50S subunit of the bacterium-like ribosome and blocking the release of peptide chains (Schlunzen et al., 2001; Schlunzen et al., 2003; Sidhu et al., 2007). The apicoplast has two indispensable roles in blood stage parasite growth: synthesis of isoprenoid precursors essential for protein prenylation, N-glycosylation, production of GPI anchors and ubiquinone biosynthesis (Yeh and DeRisi, 2011; Kennedy et al., 2019); and the phosphorylation of acetyl CoA (Swift et al., 2021). *In vitro* treatment of malaria parasites with nanomolar concentrations of azithromycin will show no growth defect within the first cycle of growth (~two days post treatment), however, progeny of treated parasites inherit a defective apicoplast and exhibit ‘delayed death’, lethally arresting parasite development during the second replication cycle (~four days post treatment) (Dahl and Rosenthal, 2007; Goodman et al., 2007). Disruption of isoprenoid biosynthesis by apicoplast ribosome targeting antibiotics results in the loss of isoprenoid-mediated protein prenylation that is required for vesicular trafficking and food vacuole formation, thereby preventing digestion of RBC haemoglobin - the major food source of the parasite (Yeh and DeRisi, 2011; Kennedy et al., 2019).

The long half-life of azithromcyin (>50 hrs) (Lode et al., 1996), its safety profile for pregnant woman and children (Hopkins, 1991), and its *in vitro* potency against *P. falciparum* have led to its promotion as an antimalarial (Andersen et al., 1998; Taylor et al., 1999; Krudsood et al., 2000; Sykes et al., 2009; Rosenthal, 2016), but the delayed death phenomenon make it unsuitable as a primary antimalarial because clearance of parasites is too slow (van Eijk and Terlouw, 2011). Other favourable traits of azithromycin include: i) improved clinical outcomes for intermittent preventative treatment for malaria in pregnancy and seasonal malaria chemoprevention in combination with drugs such as sulfadoxine-pyrimethamine (Luntamo et al., 2010; Unger et al., 2015; Chandramohan et al., 2019; Phiri et al., 2021), although these improvements can be associated with reduced disease associated with bacterial infections, ii) efficacy as a prophylactic in combination with naphthoquine, a 4-aminoquinoline, in Phase II clinical trials (Yang et al., 2018), and iii) reduced rates of *P. falciparum* infection and clinical burden as a monotherapy during mass drug administration for trachoma (Schachterle et al., 2014). Azithromycin has also been shown to inhibit liver stage development in *in vivo* rodent models of malaria and disrupt transmission of gametocytes to mosquitoes *in vitro* (Friesen et al., 2010; Shimizu et al., 2010). Thus, azithromycin has several promising antimalarial properties but has the key limiting factor of delayed death. Here, we examine a range of azithromycin analogues in the search for improved quick killing activity whilst retaining the delayed death component in pursuit of a more suitable type of azithromycin-like antimalarial.

We previously investigated a secondary, quick-killing, mechanism of action for azithromycin and select analogues (Goodman et al., 2013; Wilson et al., 2015; Burns et al., 2020). Azithromycin and analogues were demonstrated to rapidly inhibit *P. falciparum* merozoite invasion of RBCs and effectively kill asexual stages throughout one full blood stage lifecycle (rings to schizonts, in cycle, ~48 hrs). Azithromycin was equipotent throughout the entire blood stage lifecycle, and the most potent analogues were active against ring-stage parasites (<6 hrs treatments) at nanomolar potencies (Wilson et al., 2015; Burns et al., 2020), a desirable property for an antimalarial targeting asexual stages which most clinically used drugs have failed to achieve. This ‘quick-killing’ activity was active against parasites selected for resistance to the delayed death activity of azithromycin and against parasites that had their apicoplast chemically removed, confirming the quick killing mechanism to be independent of apicoplast targeted delayed death (Wilson et al., 2015; Burns et al., 2020). Given concerns that repurposing an antibiotic into an antimalarial could potentially select for azithromycin resistance in pathogenic bacteria (Lee et al., 2010) and cause dysbiosis of the human microbiome (Wei et al., 2018), medicinal chemistry synthesis efforts have been directed in making non-antibiotic azithromycin analogues (Pesic et al., 2012; Starcevic et al., 2012). These studies made use of the fact that adding an active functional group to the desosaminyl sugar of azithromycin typically abrogates binding to bacterium-like ribosomes and abolishes antibacterial activity (Pesic et al., 2012; Starcevic et al., 2012).

In efforts to overcome rising antibacterial resistance to azithromycin (Sutcliffe et al., 1996; Tait-Kamradt et al., 1997; Schroeder and Stephens, 2016) a number of studies have modified azithromycin in efforts to improve activity against macrolide resistant bacteria and broaden its antibacterial spectrum (Ma et al., 2009; Ma et al., 2010; Yan et al., 2017). Studies by Yan et al. (2017) explored the effect of substituting functional groups on the 2’ or 3’ positions of the desosaminyl sugar, as well as modifying the C11 and C12 sites on descladinosyl azithromycin against both macrolide resistant and sensitive bacteria (Yan et al., 2017). These analogues showed improved activity against bacteria encoding the macrolide resistance genes: i) erythromycin ribosomal methylase (*erm*) that modifies a specific residue within the bacterial ribosome *via* methylation and interferes with drug binding, and ii) the macrolide efflux (*mef*) gene that transports the drug out from the bacterial cell (Yan et al., 2017). However, most analogues lost potency against sensitive bacteria and were at best equivalent but not superior to azithromycin (Yan et al., 2017).

Here, we sought to address whether the azithromycin analogues represented in this panel, with their diversity of functional groups and sites of attachment, exhibited improved quick-killing and/or maintained delayed death against malaria parasites. The majority of analogues featured superior quick-killing potency over azithromycin against two different human malaria parasites and were effective at killing ring stages with <12 hrs treatment. Moreover, these drugs exhibited little in the way of apicoplast-targeting delayed death activity, suggesting that a broad range of structural modifications to azithromycin can be used to specifically improve antimalarial quick-killing activity, but activity targeting the apicoplast ribosome is not so tolerant.

## Materials and methods

### Antimalarial drugs

**Figure 1:**
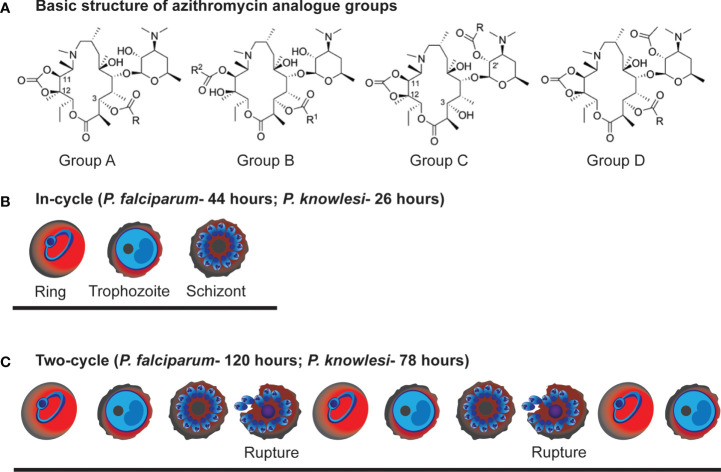
Azithromycin analogue groups and example stage progression of In-cycle and 2-cycle assays used in the study. **(A)** Basic chemical structure of the azithromycin analogue groups characterised for their antimalarial activity in this study. **(B)** In-cycle: early-ring stage parasites (0-4 hrs post-invasion) were drug treated and the resulting parasite growth measured at late schizont stage (44 hrs post-invasion for *P. falciparum* and 26 hrs for *P. knowlesi*). **(C)** early-ring stage parasites (0-4 hrs post-invasion) were drug treated and the resulting parasite growth measured at late schizont stage after 2 rupture cycles (120 hrs post-invasion for *P. falciparum* and 78 hrs for *P. knowlesi*).

Azithromycin was purchased from AK-scientific (Union City, CA, USA). Synthesis of analogues is described previously (Yan et al., 2017) with **Figure 1A** and **Supplementary Tables 1–4** providing further details of chemical structure and the origin of each analogue. Drug stocks of azithromycin (100 mM) (AK Scientific) and all analogues (10 mM) were made in ethanol. Drugs were added such that the vehicle was diluted >1000-fold for intracellular growth assays to minimise non-specific inhibition by the vehicle.

### Culture and synchronisation of *Plasmodium* spp. parasites

Green fluorescent protein (GFP) expressing *P. falciparum* (D10-PfPHG) (Wilson et al., 2010) and *P. knowlesi* (PkYHI) parasites (Lim et al., 2013) were cultured in human O^+^ red blood cells (RBCs) (Australian Red Cross Blood Service) in RPMI-HEPES culture medium (Thermo Fisher Scientific) supplemented with 0.5% v/v Albumax (Gibco), 52 μM gentamycin (Gibco), 367 μM hypoxanthine (Sigma-Aldrich), and 2 mM sodium bicarbonate (Thermo Fisher Scientific), adjusted to a pH of 7.2-7.4. Cultures were grown in sealed containers with 1% O_2_, 5% CO_2_ and 94% N_2_ (BOC Gases) at 37°C (Trager and Jensen, 1976). Synchronization of D10-PfPHG parasites for growth inhibition assays was achieved using heparin sodium (Pfizer) as previously described (Boyle et al., 2010; Wilson et al., 2013). PkYH1 parasites were passaged over a gradient of 70% Percoll (Sigma-Aldrich) to purify late stage schizonts which were then allowed to rupture for ~4 hrs in the presence of fresh RBCs prior to ring-stage treatment with 5% w/v sorbitol (Sigma-Aldrich), enabling effective synchronisation of 0-4 hrs old rings.

### Drug inhibition assays

Growth assay protocols for measuring drug inhibition of one cycle, ring to schizont stages (approximately 44 hrs post-invasion for *P. falciparum* and 28 hrs post-invasion for *P. knowlesi*) (**Figure 1B**), and two cycle (120 hrs post-invasion for *P. falciparum* and 78 hrs post-invasion for *P. knowlesi*, 2 cycles of replication) (**Figure 1C**) have been described previously (Wilson et al., 2013; Wilson et al., 2015; Lyth et al., 2018). For the assessment of stage specificity for azithromycin or analogues during the blood stage lifecycle of *P. falciparum*, each respective drug was removed at the specified time point (0-6 hrs or 0-12 hrs). To remove drug, cultures underwent three consecutive washes with 200 μl medium (centrifuged at 300 x g for 2 mins) with the final resuspension in 100 μl of fresh medium. Parasite growth at late trophozoite/schizont stages (44-48 hrs post invasion for *P. falciparum*; 24-30 hrs post invasion for *P. knowlesi*) was quantified using flow cytometry of parasites stained with ethidium bromide (EtBr) (10 μg/mL for 1 hr) prior to washing with PBS.

### Apicoplast-null inhibition assays

Apicoplast null (D10-PfPHG^apicoplast-null^) parasites were generated as previously described (Yeh and DeRisi, 2011; Uddin et al., 2018). Briefly, the culture medium was supplemented with 200 μM isopentenyl pyrophosphate (IPP) (NuChem Therapeutics, Canada) and 0.35 μM (5x IC_50_) of azithromycin for a minimum of 6 days (~three cycles) and parasites were cultured continuously thereafter with IPP. Successful removal of the apicoplast was assessed by growing D10-PfPHG^wildtype^ and D10-PfPHG^apicoplast-null^ parasites with reducing concentrations of azithromycin for ~120 hrs (delayed death), which confirmed a loss of sensitivity to azithromycin as observed by a ~64 fold-change in the IC_50_ with apicoplast removal (D10-PfPHG^apicoplast-null^ IC_50_, 4.5 μM; D10-PfPHG^wildtype^ IC_50_, 0.07 μM) (**Supplementary Figure 1**). To assess the inhibitory activity of analogues, D10-PfPHG^apicoplast-null^ and D10-PfPHG^wildtype^ parasites were grown in the presence of the IC_90_ obtained for D10-PfPHG^wildtype^ parasites in in cycle (0-44 hrs) or delayed death (0-120 hrs) assays, or a dilution series of the respective drug. Drugs were added to tightly synchronised ring stage D10-PfPHG^apicoplast-null^ (+ 200 μM IPP) or D10-PfPHG^wildtype^ (no IPP) parasite lines and assays were incubated for ~44 hrs (in-cycle) or 120 hrs (two cycle delayed death) as specified with the resulting parasitemia quantitated by flow cytometry.

### Flow cytometry and microscopy analysis of growth inhibition

Parasitaemia was measured on an LSR Fortessa (Becton Dickinson) using a 96-well plate reader. Mature (>36 hrs post-invasion) *P. falciparum* D10-PfPHG parasites were counted using Fl-1-high (GFP; excitation wavelength, 488 nm) and Fl-2-high (EtBr; excitation wavelength, 488 nm) (Wilson et al., 2013). Mature parasites of the PkYH1 line (>24 hrs post-invasion) were gated with a forward scatter (FSC) and FL-2-high (EtBr) gate. Typically, 20,000-40,000 RBCs were counted in each well. All samples were analysed using FlowJo software (TreeStar Inc, Ashland, OR, USA) and growth of drug treatments were normalised against growth of media control wells to calculate the percent survival of drug treated parasites. To address the phenotypic effects of drugs, thin smears were fixed with fresh methanol and stained in fresh 10% Giemsa (Merck) for 10 mins before images of drug treated parasites were taken with an Olympus BX51/BX52 light microscope with immersion oil using 100X magnification.

### Toxoplasma gondii drug assay

Growth assays for drug inhibition of *Toxoplasma gondii* were conducted using RH-strain parasites expressing yellow fluorescent protein (Gubbels et al., 2003). Freshly-lysed parasites were used to infect HFF monolayers at 2x10^3^ parasites/well in 96-well plates, in FluoroBrite DMEM culture medium (Invitrogen) supplemented with 10% FCS. Drugs were added and plates were incubated at 37°C in 5% CO_2_. At completion of the incubation periods (72 hrs and 120 hrs), fluorescence read-outs were obtained using the BMG LabTech PHERAstar FS microplate reader set at an excitation of 485 nm and emission of 520 nm.

### Mammalian cell cytotoxicity

Toxicity against mammalian cells was determined using the Huh-7D cell line derived from human hepatocellular carcinoma cells (Sigma-Aldrich). Huh-7D cells were maintained in Dulbecco’s Modified Eagle Medium (DMEM) (Gibco, Thermo Fisher Scientific) supplemented with 10% fetal bovine serum (FBS) and non-essential amino acids. Huh-7D cells were grown in an atmosphere of 5% CO_2_ in a 37°C incubator. Cultures were seeded to 40,000 cells in round bottom 96-well microtiter plates (Corning) and incubated with two-fold serial dilutions of drug for 24 hrs in 5% CO_2_ at 37°C. Post incubation 1:1 addition of CellTiter-Glo Reagent (Promega, USA) was added to each well to lyse HuH-7D cells and release ATP for detection by luminescence (Posimo et al., 2014). Plates were incubated for 10 mins to allow the luminescent signal to stabilise, which was then detected using a Phera Star FS using the luminescent module (Lum Plus, spectral wavelength 230 nM to 750 nM). Cell viability with drug treatment was assessed by comparing cell replication in drug treated wells and normalizing this growth against non-inhibitory control wells (media).

### Statistical analysis

All IC_50_, IC_90_ and cytotoxicity concentration (CC_50_) estimates were determined using GraphPad Prism (GraphPad Software) according to the recommended protocol for nonlinear regression (constrained to top= 100 and bottom= 0) of a log-(inhibitor)-versus-response curve. Statistical significance between drug treatments were determined with the GraphPad Prism software using the log-(inhibitor)-versus-response curve with Extra Sum-of-Squares F Test (best-fit LogIC_50_). *P* values were considered significant if P=<0.05.

### Antibacterial screen

Minimum inhibitory concentration (MIC) assays for assessment of *Streptococcus pneumoniae* sensitivity to azithromycin and analogues were performed as described (Wiegand et al., 2008). Briefly, the antibacterial activity of azithromycin and all analogues were assessed with a two-fold serial dilution in the presence of macrolide sensitive *S. pneumoniae* D39. Cells were inoculated at a final concentration of approximately 10^6^ CFU/mL in Mueller Hinton Broth supplemented with 5% lysed horse blood. The MIC was determined to be the concentration of drug that inhibited bacterial growth within a 96-well microtiter tray after 24 hrs incubation at 37°C. Drug activity was assessed by determining the minimal inhibitory concentration (MIC) that stopped bacterial growth, as indicated by a media colour change. MICs are expressed as µM.

### Sample extraction for metabolomics analysis

For metabolomics experiments, two 150 mL flasks at 6% haematocrit containing tightly synchronised parasites 28-34 hrs post-invasion (5-6 hrs rupture window), were harvested *via* magnet purification (Miltenyi Biotech). Infected RBC density was quantitated by flow cytometry (Tham et al., 2010) and 2 mL of 3x 10^7^ parasites were added into the wells of 24 well microtiter plates. Parasites were incubated for 1 hrs at 37°C to stabilise the culture. Following this initial incubation, 5x IC_50_ of the azithromycin analogue C1, the control drugs chloroquine, dihydroartemisinin (DHA) and azithromycin, and the vehicle control ethanol were added and incubated for 2 hrs. Supernatant was removed and parasites washed twice with 800 μL ice-cold 1 x PBS, with cells pelleted *via* centrifugation at 400 x gs for 5 mins at 0°C. Cell pellets were resuspended in 200 μL of ice-cold extraction buffer (CHCl_3_/MeOH/water (1:3:1 *v/v*)) containing 1 µM internal standards, CHAPS and PIPES, and then incubated on ice for 1 hrs with shaking at 200 rpm. Cell debris was pelleted with centrifugation at 14800 x gs for 10 mins at 0°C. The resulting supernatant (180 µL) was transferred to Eppendorf tubes and the remaining ~20 µL were combined to make a pooled QC sample. Extraction blank samples (without cells) were prepared alongside and samples were stored at -80°C until analysis.

### LC-MS analysis

Liquid chromatography-mass spectrometry (LC-MS) data was acquired on a Q-Exactive Orbitrap mass spectrometer (Thermo Scientific) coupled with high-performance liquid chromatography system (HPLC, Dionex Ultimate^®^ 3000 RS, Thermo Scientific) as previously described (Creek et al., 2016). Briefly, chromatographic separation was performed on ZIC-pHILIC column equipped with a guard (5 µm, 4.6 × 150 mm, SeQuant^®^, Merck). The mobile phase (A) was 20 mM ammonium carbonate (Sigma Aldrich), (B) acetonitrile (Burdick and Jackson) and needle wash solution was 50% isopropanol. The column flow rate was maintained at 0.3 mL/min with temperature at 25°C and the gradient program was as follows: 80% B decreasing to 50% B over 15 min, then to 5% B at 18 min until 21 min, increasing to 80% B at 24 min until 32 min. Total run time was 32 min with an injection volume of 10 µL. The mass spectrometer operated in full scan mode with positive and negative polarity switching at 35 k resolution at 200 *m/z* with detection range of 85 to 1275 *m/z*, AGC target was 1e^6^ ions, maximum injection time 50 ms. Electro-spray ionization source (HESI) was set to 4.0 kV voltage for positive and negative mode, sheath gas was set to 50, aux gas to 20 and sweep gas to 2 arbitrary units, capillary temperature 300°C, probe heater temperature 120°C. The samples were analyzed as a single batch to avoid batch-to-batch variation and randomized to account for LCMS system drift over time.

### LC-MS metabolomics data processing

The acquired LCMS data was processed in untargeted fashion using the open source software, IDEOM (Creek et al., 2012) (http://mzmatch.sourceforge.net/ideom.php). Initially, *ProteoWizard* was used to convert raw LC-MS files to *mzXML* format and *XCMS* (Centwave) to pick peaks. *Mzmatch.R* was used to convert to *peakML* files, align samples and filter peaks using minimum detectable intensity of 100,000, relative standard deviation (RSD) of <0.5 (reproducibility), and peak shape (codadw) of >0.8. *Mzmatch* was also used to retrieve missing peaks and annotation of related peaks. Default IDEOM parameters were used to eliminate unwanted noise and artefact peaks. Loss or gain of a proton was corrected in negative and positive ESI mode, respectively, followed by putative identification of metabolites by accurate mass within 3 ppm mass error searching against the Kyoto Encyclopedia of Genes and Genomes (KEGG), MetaCyc, HMDB and LIPIDMAPS databases and a theoretical database of short peptides. To reduce the number of false positive identifications, retention time error was calculated for each putatively identified metabolite using IDEOM’s built-in retention time model which is based on actual retention time data of authentic standards (~350 standards). Statistical analysis on filtered data was performed using the Matboanalyst web interface (Chong et al., 2018).

## Results

### Azithromycin analogues with diverse modifications have improved quick-killing activity against malaria parasites *in vitro*

All 22 azithromycin analogues (structures available in **Supplementary Tables 1–4**) were initially assessed for growth inhibition at 10 μM with *in vitro* in cycle assays (**Figure 1B**), from early rings to early schizonts, against *P. falciparum* (D10-PfPHG, 44 hrs) (Wilson et al., 2010) or *P. knowlesi* (PkYH1, 28 hrs) (Lim et al., 2013) [in cycle (**Supplementary Tables 1–4**)]. This primary screen identified 17 of 22 analogues for *P. falciparum* (growth inhibition of between 98% to 69% for these 17 compounds) and 18 analogues for *P. knowlesi* (growth inhibition of between 99% to 76% for these 18 compounds) that inhibited growth by >40% under these conditions. The 17 analogues inhibitory against *P. falciparum* also inhibited *P. knowlesi* and one analogue (A4) that was active in *P. knowlesi* but not *P. falciparum* were all prioritised for further evaluation.

The in cycle IC_50_ values for the 17 and 18 analogues identified from primary screens were then determined against D10-PfPHG and PkYH1 lines, respectively. All analogues showed improved quick-killing IC_50_ values compared to azithromycin (azithromycin in cycle IC_50_- *P. falciparum* 11 μM; *P. knowlesi* 13 μM). There was a 1.6 to 30-fold improvement in quick-killing activity against *P. falciparum* with four analogues showing >10-fold greater potency than azithromycin (IC_50_; A13, 0.72 μM; B2 0.56 μM; C1, 0.33 μM; and D1, 0.67 μM) (**Table 1** and **Supplementary Table 5**). There was a 1.7 to 20-fold improvement in quick-killing potency against *P. knowlesi* (PkYH1) with five analogues demonstrating >10-fold activity compared to azithromycin (IC_50_; A2, 0.67 μM; A3, 1.11 μM; A8, 0.81 μM; A13, 0.64 μM; B2, 0.69 μM) (**Table 1** and **Supplementary Table 5**).

**Table 1:** *In vitro* efficacy of azithromycin analogues against *Plasmodium* parasites.

Compound	In-cycle growth μM(44 hr IC_50_) D10-PfPHG ^a^	In-cycle growth μM (28 hr IC_50_) PkYH1^a^	Delayed death μM (120 hr IC_50_) D10-PfPHG^b^	Delayed death μM (96 hr IC_50_) PkYH1^b^
Azithromycin	10 (*1.1*)	13 (*1.8*)	0.02 (*0.01*)	0.09 (*0.02*)
A2	1.6 (*0.2*)	0.67 (*0.1*)	0.26 (*0.06*)	0.24 (*0.04*)
A3	1.5 (*0.07*)	1.1 (*0.3*)	0.47 (*0.09*)	0.36 (*0.05*)
A4	ND	7.8 (*1.0*)	ND	ND
A5	1.8 (*0.3*)	1.7 (*0.5*)	0.39 (*0.06*)	0.43 (*0.04*)
A7	5.3 (*0.2*)	1.8 (*0.1*)	0.76 *(0.02)*	0.54 *(0.08)*
A8	1.4 (*0.2*)	0.81 (*0.2*)	0.36 (*0.05*)	0.14 (*0.01*)
A9	3.6 (*0.6*)	2.0 (*0.5*)	0.82 (*0.08*)	0.69 (*0.08*)
A10	4.2 (*0.5*)	1.5 (*0.3*)	1.1 *(0.03)*	0.66 (*0.1*)
A11	6.1 (*0.6*)	4.5 (*0.9*)	0.92 *(0.01)*	0.89 (*0.3*)
A12	1.8 (*0.8*)	1.3 (*0.08*)	0.32 (*0.03*)	0.19 (*0.02*)
A13	0.72 (*0.01*)	0.6 (*0.02*)	0.18 (*0.02*)	0.39 (*0.03*)
B1	1.6 (*0.4*)	1.3 (*0.2*)	0.29 (*0.02*)	0.1 (*0.03*)
B2	0.56 (*0.05*)	0.69 (*0.1*)	0.14 (*0.01*)	0.09 (*0.03*)
C1	0.33 (*0.02*)	1.4 (*0.2*)	0.12 (*0.01*)	0.27 (*0.1*)
C2	4.6 (*0.5*)	5.9 (*1.9)*	1.1 *(0.14)*	ND
C3	3.2 (*0.2*)	3.9 (*0.6*)	0.99 *(0.09)*	ND
D1	0.67 (*0.04*)	1.7 (*0.3*)	0.12 *(0.03)*	0.74 *(0.4)*
D2	3.7 (*0.3*)	2.9 (*0.4*)	1.3 *(0.01)*	ND

a Quick-killing in-cycle drug treatment from rings to late schizonts, with no rupture cycle (D10-PfPHG, *P. falciparum*, 0-44 hrs or PkYH1, *P. knowlesi* 0-28 hrs).

b Delayed death drug treatment, from early-rings to late trophozoites, with two rupture cycle (D10-PfPHG, *P. falciparum*, 0-120 hrs or PkYH1, *P. knowlesi* 0-78 hrs).

Data represents the means of 2 (or more) experiments expressed as percentage of non-inhibitory control.

ND= not done due to limited sample.

### Diverse azithromycin analogues have improved activity against ring stage parasites

**Figure 2:**
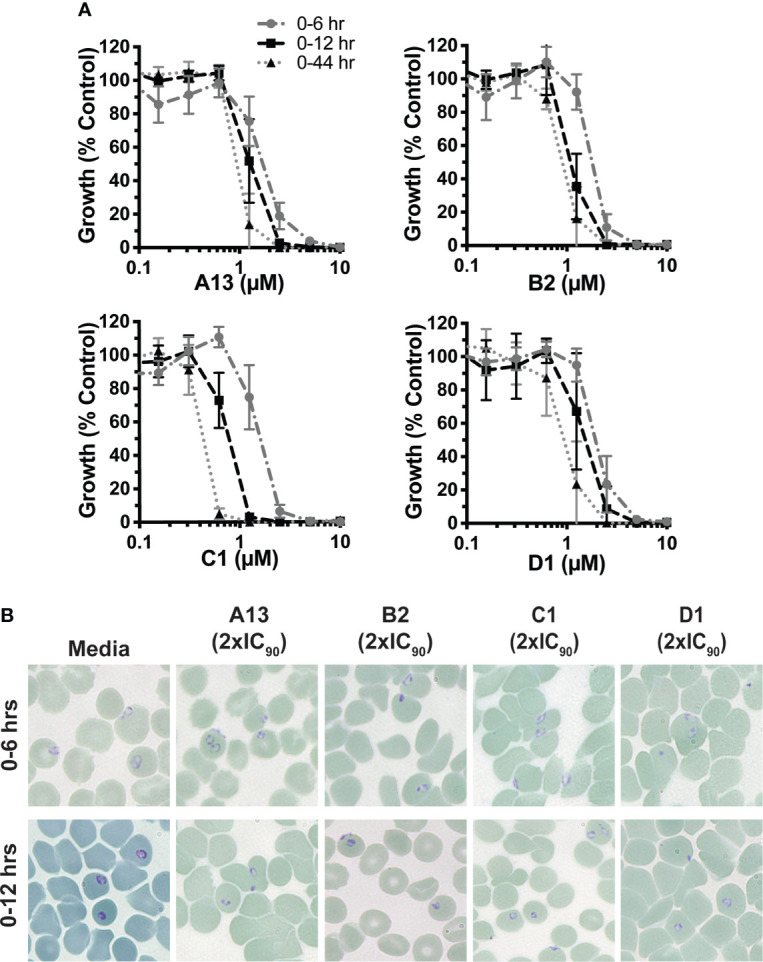
Growth inhibition profiles of azithromycin analogues with short term treatment **(A)** Growth inhibition profile of A13, B2, C1 and D1 with very early ring-stage treatment across 0-6 hrs and 0-12 hrs post-invasion compared to a full in-cycle treatment. Early ring-stage *P. falciparum* (D10-PfPHG) parasites (<4 hrs post-invasion) were treated with doubling dilutions of azithromycin analogues for 0-6 hrs or 0-12 hrs prior to washing the drug out, with cultures allowed to continue growing until parasites were 44 hrs old. A 0-44 hrs control where parasites were maintained on drug was also included. There were significant differences between all 0-6 hrs vs 0-44 hr treatments of A13, B2, C1 and D1 (A13 and B2 P=0.01; C1 and <0.0001). A significant difference was also observed for 0-6 hrs vs 0-12 hrs of A13, B2 and C1(A13 P=0.01; C1 and B2 P=<0.0001) but not D1 (P=NS). Significant difference was observed for 0-12 hrs vs 0-44 hrs for C1 and D1 (P= 0.01), but not A13 or B2 (P=NS). Parasitemia was measured *via* flow cytometry 44 hrs post-invasion. Data represents the means of 3 or more experiments expressed as a percentage of non-inhibitory control and error bars represent SEM. **(B)** Representative Giemsa stained thin blood smears showing the growth phenotypes seen for non-inhibitory media controls, azithromycin analogues A13, B2, C1 and D1 (2 x IC_90_) 0-6 hrs post treatment (top panels) and 0-12 hrs post treatment (bottom panel). Data for parallel azithromycin and dihydroartemisinin treatments are available in (Burns et al., 2020).

Recently, we showed that azithromycin and analogues are active against early ring stage development (<12 hrs of treatment) as well as broadly inhibitory throughout the blood stage lifecycle with ~12 hrs treatment intervals (Burns et al., 2020). Such broad activity is of interest for clinical treatment as most antimalarials exhibit limited efficacy against early rings (Zhang et al., 1986; Cowman et al., 1988; Wilson et al., 2013). To assess whether this held true for the analogues tested in this study, early ring stage D10-PfPHG parasites (0-4 hrs post invasion) were treated for 6 hrs and 12 hrs with the analogues showing the highest in cycle potency against *P. falciparum* (A13, B2, C1 and D1). All four analogues exhibited activity against both early (0-6 hrs) and late (0-12 hrs) ring stage treatments with a higher potency seen for longer 12 hrs treatments (A13 (IC_50_; 0-6 hrs, 2.3 μM; 0-12 hrs, 1.4 μM; 0-44 hrs, 0.72 μM), B2 (IC_50_; 0-6 hrs, 2.8 μM; 0-12 hrs, 1.4 μM; 0-44 hrs, 0.56 μM), C1 (IC_50_; 0-6 hrs, 1.5 μM; 0-12 hrs, 0.7 μM; 0-44 hrs, 0.33 μM) and D1 (IC_50_; 0-6 hrs, 2.1 μM; 0-12 hrs, 1.5 μM; 0-44 hrs, 0.67 μM)) (**Figure 2A**). For analogues A13, B2, C1 and D1 the IC_50_ of 12 hrs treatment differed <3-fold in comparison to the IC_50_ of 44 hrs treatment. Shorter 6 hrs ring stage treatments exhibited between a 3.1 and 5-fold rise in IC_50_ over 44 hrs treatments, however, the activity of these analogues with a 6 hrs treatment was still significantly better than that recorded for azithromycin (IC_50_; 0-6 hrs, 30 μM; 0-12 hrs, 16 μM) (Burns et al., 2020).

We next examined the effect of 6 hrs and 12 hrs ring stage drug treatments on parasite morphology at a 2 x IC_90_ concentration (0-44 hrs) for the most potent analogues A13, B1, C1 or D1 using light microscopy (**Figure 2B**). No aberrant growth phenotype was obvious for 6 hrs treatments of early ring stage parasites with any drug. Examination of 12 hrs treatments showed evidence of underdeveloped parasites for all four azithromycin analogues, indicating parasite stress in the face of drug pressure. These data demonstrate that the most potent analogues have activity against early ring stage parasite growth.

### Activity against the related apicomplexan parasite *Toxoplasma gondii* and toxicity against human Huh-7D cells

Previously, we showed that azithromycin and analogues have moderate invasion inhibitory activity against *Toxoplasma gondii* (Wilson et al., 2015). We tested whether our most potent analogues also displayed evidence of improved quick-killing activity against *T. gondii*. Compounds A13 (IC_50_ 2.3 μM), B2 (IC_50_ 1.4 μM), C1 (IC_50_ 3 μM) were 3, 2.5 and 10-fold less potent with 72 hrs *T. gondii* treatment than for 44 hrs *P. falciparum* treatment. Further, they all exhibited lower potency than azithromycin (IC_50_ 0.5 μM) with 72 hrs treatment, indicating that the analogues have minimal quick-killing activity against *T. gondii*.

Azithromycin and analogues previously found to have quick killing activity (Burns et al., 2020) have regularly been reported to have cytotoxicity levels *in vitro* (CC_50_) of >10 μM, and often show no toxicity to mammalian cells at >50 μM (Bukvic Krajacic et al., 2011; Peric et al., 2012; Peric et al., 2021). Therefore, we next investigated the potential of mammalian cell cytotoxicity for a focused group of analogues on the human hepatocellular carcinoma, Huh-7D, cell line (McKim, 2010). Inhibition of Huh-7D cell growth for analogues featuring an IC_50_ of <1 μM in either D10-PfPHG or PkYH1 parasite lines (A2, A8, B2 and C1) was assessed using an ATP-based luminesce detection assay (Posimo et al., 2014). Two analogues, A13 and D1, with IC_50_ values of <1 μM were excluded from this analysis due to limited sample. Compounds A2, A8 and B2 had cytotoxicity levels *in vitro* (CC_50_) of >8.5 μM with >5-fold and >10-fold higher selectivity index in comparison to the IC_50_s observed for D10-PfPHG or PkYH1, respectively (**Table 2**). The selectivity index of C1 (CC_50_, 2.6 μM) was 7.9 for *P. falciparum* (IC_50_, 0.3 μM) but dropped to 1.6 for *P. knowlesi.*

**Table 2:** Cytotoxicity of lead analogues and selectivity against *Plasmodium* spp. Parasites.

Compound	Intracellular growth μM(44 hr IC_50_) D10-PfPHG ^a^	Intracellular growth μM (28 hr IC_50_) PkYH1^a^	Cytotoxicity μM (CC_50_) Huh-7D^c^	Selectivity Huh-7D (CC_50_)/PfPHG IC_50_ ^d^	Selectivity Huh-7D (CC_50_)/PkYH1IC_50_ ^d^
A2	1.6 (*0.2*)	0.67 (*0.13*)	9.2 (*0.9*)	5.8	13.7
A8	1.4 (*0.2*)	0.81 (*0.2*)	9.9 (*1.2*)	7	12.2
B2	0.56 (*0.05*)	0.69 (*0.1*)	8.5 (*1.4*)	15.1	12.3
C1	0.33 (*0.02*)	1.4 (*0.2*)	2.6 (*0.2*)	7.9	1.9

a Quick-killing in-cycle drug treatment from rings to late schizonts, with no rupture cycle (D10-PfPHG, *P. falciparum*, 0-44 hrs or PkYHI, *P. knowlesi* 0-28 hrs).

c Drug treatment of Huh-7D cells were incubated with the compounds for 24 hrs before the viability was measured. Data represents the means of 3 (or more) experiments expressed as percentage of non-inhibitory control.

d Selectivity index is calculated as the cytotoxic concentration 50 (CC_50_) for Huh-7D cells divided by the inhibitory concentration 50 (IC_50_) against *Plasmodium* spp parasites.

### Metabolomic investigation of the most potent compound

We next used an untargeted metabolomics approach to identify changes in the metabolomic signatures of late trophozoites stages treated for 2 hrs at 5x IC_50_ (44 hr) with the most potent analogue in *P. falciparum*, namely C1. Few metabolic pathways were impacted by C1, with the major impact being upregulation of a range of peptides, consistent with the profile observed for chloroquine and azithromycin (**Supplementary Table 6**). Interestingly, most of these peptides are not derived from haemoglobin (the primary source of peptides in the acidic digestive vacuole of the parasite), and the source of these peptides cannot be determined due to their short sequence length. Nevertheless, this metabolic signature of elevated non-haemoglobin-derived peptides has been reported before for 4-aminoquinolines such as chloroquine and closely related analogues, suggesting that this profile is a biomarker of chloroquine-like activity in the parasite (Creek et al., 2016; Birrell et al., 2020). C1 also upregulated some unique peptides that were not impacted by azithromycin and chloroquine (**Supplementary Table 7**), which may be derived from haemoglobin. Overall, the metabolomics profile supports our earlier proposition that azithromycin and many analogues have broad activity against trophozoite stage parasites, including the food vacuole (Burns et al., 2020).

### Activity of azithromycin analogues against the bacterium-like apicoplast ribosome

After identifying azithromycin analogues with improved quick-killing activities against the blood stages of malaria, we next assessed apicoplast-targeting delayed death activity for each analogue. Given that the delayed death IC_50_ of azithromycin is 0.02 μM for *P. falciparum* and 0.09 μM for *P. knowlesi* parasites, we reasoned that the delayed death activity of analogues would most likely be evident at drug concentrations below 1 μM across 2-cycles of parasite growth. All 22 analogues were screened for potential delayed death activity by treating *P. falciparum* (120 hrs treatment) and *P. knowlesi* (78 hrs treatment) parasites with 1 μM of drug and quantifying growth inhibition after two rupture cycles. This screen identified 10 of 22 analogues that inhibited growth by >30% at 1 μM across 2-cycles in both *Plasmodium* spp., with two analogues, A10 and A11, only active against *P. knowlesi* (**Supplementary Tables 1**-**4**). We then evaluated the IC_50_s for these prioritised drugs across 2-cycles of parasite growth in the respective *Plasmodium* spp. The majority of analogues featured high-nanomolar activity against *P. falciparum* across 2-cycles of parasite growth and were between 6 to 140-fold less potent (IC_50_ range; C4, 2.8 μM to C1 and D1, 0.12 μM) than azithromycin (0.02 μM) (**Table 1**; **Supplementary Table 8**). Similarly, the analogues tested against *P. knowlesi* showed 1.1 to 9.9-fold higher IC_50_s (IC_50_ range; A11, 0.89 μM to B2, 0.09 μM) than azithromycin (0.09 μM) (**Table 1**; **Supplementary Table 8**). Notably, the analogues showing a 2-cycle IC_50_ similar to azithromycin were also the most potent in 44 hr treatments, opening up the possibility that the improved activity over 120 hrs treatments was due to the cumulative activity of quick-killing across two growth cycles.

**Figure 3:**
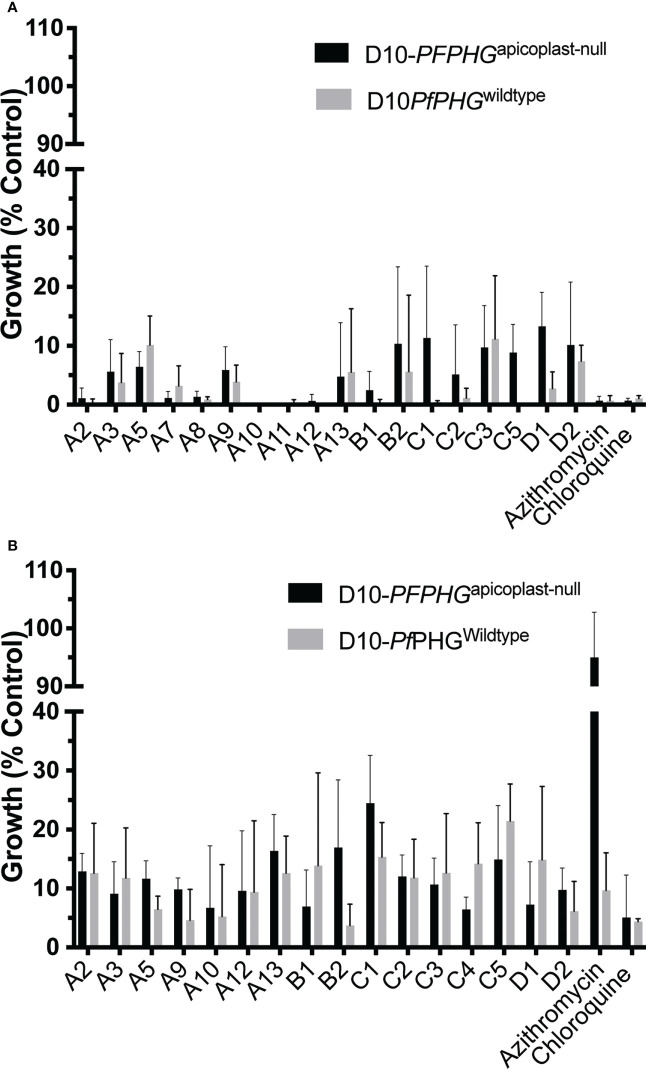
Activity of azithromycin analogues in the presence or absence of the apicoplast. Early ring-stage D10-PfPHG^wildtype^ (no IPP) (grey bars) or D10-PfPHG^apicoplast-null^ (+IPP) (black bars) *P. falciparum* parasites (0-4 hrs post-invasion) were treated with: **(A)** the IC_90_ of in cycle growth inhibition, addition of IPP did not rescue parasite growth from quick-killing activity of azithromycin or analogues, and **(B)** the IC_90_ of 2 cycle (delayed-death) growth inhibition, IPP rescued parasite growth from azithromycin’s delayed death activity (inhibitor of the apicoplast’s bacterial-like ribosome) but did not rescue parasite growth from chloroquine (targets the digestive vacuole) or analogues. Parasitemia was measured at 44 hr or 120 hrs post invasion at schizont stage *via* flow cytometry for in cycle and delayed death assays, respectively. Data represents the means of 3 (or more) experiments expressed as percentage of non-inhibitory control and error bars represent the ± SEM.

To address whether the improved activity over 2-cycles of parasite growth was due to quick-killing activity or apicoplast targeted delayed-death activity, the 2-cycle growth inhibitory activity for a panel of analogues was tested against parasites lacking the apicoplast (apicoplast-null) (Yeh and DeRisi, 2011; Uddin et al., 2018). First, the apicoplast was chemically removed and parasite growth rescued with IPP supplementation (Uddin et al., 2018). We next examined whether the quick-killing (in cycle, 44 hrs) activity of analogues was affected by removal of the apicoplast by treating D10-PfPHG^apicoplast-null^ and D10-PfPHG^wildtype^ lines with the in cycle D10-PfPHG^wildtype^ IC_90_ concentration of all quick-killing analogues (**Table 1**). As growth inhibition for D10-PfPHG^apicoplast-null^ parasites was comparable to D10-PfPHG^wildtype^ parasites for all drugs tested, this supported our previous observation that the quick-killing mechanism of the analogues is unrelated to apicoplast ribosome inhibition Burns et al. (2020) (**Figure 3A**; **Supplementary Table 9**).

**Figure 4:**
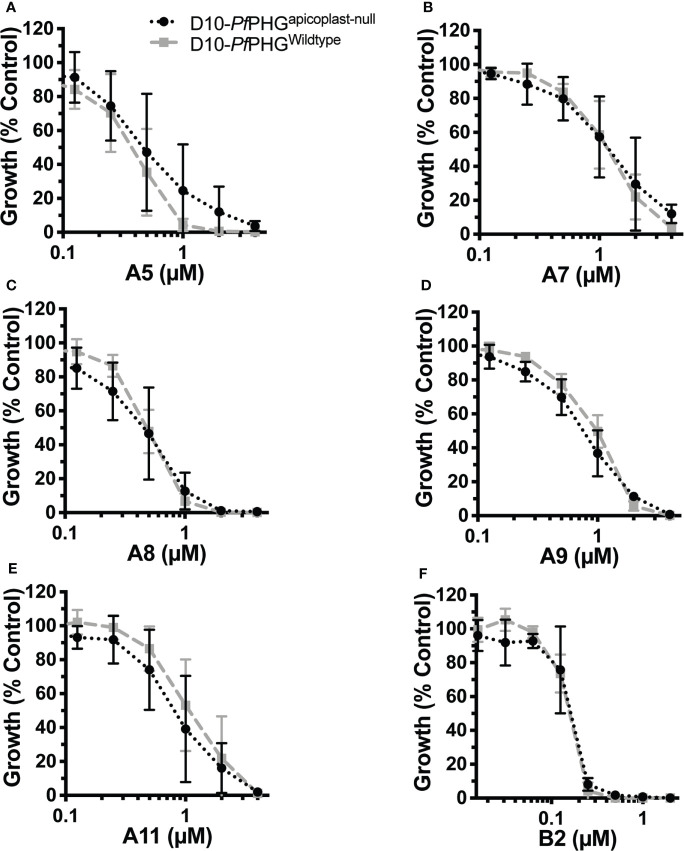
Removal of the apicoplast does not rescue parasites from azithromycin analogue activity. Early ring-stage *P. falciparum* D10-PfPHG^wildtype^ and D10-PfPHG^apicoplast-null^ parasites (<4 hrs post-invasion) were treated with doubling dilutions of analogues and inhibition of growth measured 2 cycles latter (delayed death, 120 hrs) for compounds: **(A)** A5 (D10-PfPHG^apicoplast-null^ IC_50_, 0.5 μM; D10-PfPHG^wildtype^ IC_50_, 0.4 μM. P=NS). **(B)** A7 (D10-PfPHG^apicoplast-null^ IC_50_, 0.37 μM; D10-PfPHG^wildtype^ IC_50_, 0.49 μM. P=NS). **(C)** A8 (D10-PfPHG^apicoplast-null^ IC_50_, 0.63 μM; D10-PfPHG^wildtype^ IC_50_, 0.62 μM. P=NS). **(D)** A9 (D10-PfPHG^apicoplast-null^ IC_50_, 1.4 μM; D10-PfPHG^wildtype^ IC_50_, 1.8 μM. P=NS). **(E)** A11 (D10-PfPHG^apicoplast-null^ IC_50_, 1.6 μM; D10-PfPHG^wildtype^ IC_50_, 2.9 μM. P=NS). **(F)** B2 (D10-PfPHG^apicoplast-null^ IC_50_, 0.17 μM; D10-PfPHG^wildtype^ IC_50_, 0.16 μM. P=NS). Data represents the means of 2 (or more) experiments expressed as percentage of non-inhibitory control and error bars represent the SEM.

To determine whether analogues could also kill parasites through targeting the bacterium-like ribosome of the apicoplast, we compared growth of D10-PfPHG^wildtype^ and D10-PfPHG^apicoplast-null^ lines across 2-cycle assays with the delayed death (120 hrs treatment) D10-PfPHG^wildtype^ IC_90_ concentration of azithromycin and analogues (**Table 1**). D10-PfPHG^wildtype^ parasite growth was abolished when treated with azithromycin, whereas D10-PfPHG^apicoplast-null^ parasites grew normally, consistent with the ‘rescue’ phenotype expected with an apicoplast ribosome targeting drug (Yeh and DeRisi, 2011; Uddin et al., 2018) (**Figure 3B**). However, no rescue was observed for the D10-PfPHG^apicoplast-null^ line with any analogue, results that are consistent with the activity of the non-apicoplast targeting control drug, chloroquine (targets haem-detoxification) (**Figure 3B**; **Supplementary Table 9**). To further confirm this observation, we determined the IC_50_s for analogues that exhibited micromolar quick-killing activities but nanomolar delayed death IC_50_ values (A5, A7, A8, A9, A11 and B2), a pattern similar to that of azithromycin, against D10-PfPHG^wildtype^ and D10-PfPHG^apicoplast-null^ parasites (**Table 1**). The IC_50_s of these six analogues against the D10-PfPHG^wildtype^ and D10-PfPHG^apicoplast-null^ lines were almost identical (**Figure 4**), confirming that apicoplast targeting delayed-death activity does not contribute to the activity of these compounds.

### Activity of azithromycin analogues against bacterial ribosomes

The azithromycin analogues examined in this study featured a range of modifications that could contribute to improved quick-killing potency, however, we identified that these analogues also lost delayed-death activity. While the activity of these analogues have been addressed previously using a number of macrolide resistant *Streptococcus pneumoniae* strains, these studies did not test the analogues activity against an erythromycin sensitive *S. pneumoniae* (Yan et al., 2017), thus precluding direct comparison to our earlier study that assessed quick-killing analogue activity against an erythromycin sensitive strain of *S. pneumoniae* (Burns et al., 2020). Therefore, to allow clearer comparison of whether analogues with quick-killing antimalarial activity had lost or reduced activity against a bacterial ribosome we tested the potency of all 22 analogues against the gram-positive, azithromycin sensitive, bacteria *S. pneumoniae* (D39) (**Supplementary Table 9**). However, none of the analogues showed growth inhibitory activity against *S. pneumoniae* that was equivalent to azithromycin (MIC 0.125 μM) (**Supplementary Table 9**). As the intact desosamine sugar is required for binding to the bacterial-like ribosome (Schlunzen et al., 2001; Schlunzen et al., 2003), it was no surprise that Group C and D compounds (**Supplementary Tables 3, 4**), featuring a desosamine modification, showed limited evidence of targeting bacterium-like ribosomes in this bacterial strain (MICs >4 μM). Group A, featured a cyclic carbonate in the 11, 12 positions and aryl or alkyl carbamoyl substitution in the 3-position of descladinosylazithromycin, and B analogues, featuring aryl or alkyl carbamoyl substitution of both the 3- and the 11- position of descladinosylazithromycin, also showed minimal growth inhibition after treating *S. pneumoniae* with MICs >1 μM. The only exception in this series was B1 that featured high-nanomolar activity (MIC, 0.25 μM), suggesting this analogue may retain some activity against bacterial ribosomes or has non-specific activity against the bacteria since it was not active against the ribosome of the apicoplast (**Figure 3B**; **Supplementary Table 9**).

## Discussion

Recently, we demonstrated that azithromycin and related macrolides that inhibit translation by the bacterium-like ribosomes of the malaria apicoplast and cause ‘delayed death’ of the second-generation of daughter parasites (Sidhu et al., 2007; Dahl and Rosenthal, 2007; Goodman et al., 2007) also kill blood stage parasites though ‘quick-killing’ and that chemical modification improves this activity (Wilson et al., 2015; Burns et al., 2020). Here, we characterised a panel of azithromycin analogues, previously demonstrated to have moderate efficacy against bacteria (Yan et al., 2017) for their inhibitory activity against malaria parasites. Twenty-two azithromycin analogues were assessed for quick-killing and delayed death activities against *P. falciparum* and *P. knowlesi*. We identified 17 of 22 analogues with improved in cycle potency against *P. falciparum* and 18 showing improved quick-killing (28 hrs treatment) for *P. knowlesi*, in comparison to azithromycin. Improvements in quick-killing were evident across a range of chemotypes and the activity of analogues investigated in this study was similar for both malaria *Plasmodium* species. Descladinosylazithromycin analogue C1 (3-*O*-(3-chlorophenyl) carbamoyl) was found to have the most potent quick-killing activity against *P. falciparum*. Whereas descladinosylazithromycin analogue A2 (3-*O*-(3-methylphenyl) carbamoyl), A13 (3-*O*-(phenyl) carbamoyl) and B2 (11-O-(hexyl) carbamoyl, 3-O-(3-methoxyphenyl) carbamoyl) were the most potent quick-killing analogues against *P. knowlesi.* We have previously identified that loss of azithromycin’s cladinose sugar results in a 6.5-fold reduction in potency against malaria parasites (Wilson et al., 2015). However, in this study improved quick-killing activity of analogues was evident even for those that had functionality substituted in place of the cladinose sugar. In general, due to the small size of the analogue study set, the limited structural diversity in the molecular framework and peripheral substitutions, there was no clear structural modification or functional group commonality between the most active compounds that can be exploited to prioritise further development of their quick-killing activity.

Previously, we showed azithromycin analogues to have remarkably rapid potency against early ring-stage parasites with 6 and 12 hrs treatments compared to treatment across one full cycle of intracellular growth (0-44 hrs) (Burns et al., 2020). While the compounds described in this study did not reach the potency of several analogues we described previously (Burns et al., 2020), the most potent analogues showed a clear growth inhibitory phenotype against early ring stage parasites, with underdeveloped parasites visible with 12 hrs of treatment. The growth inhibitory profile with treatment at early ring stages (0-6 hours post invasion) and later rings stages (0-12 hours) was most similar in profile to what we found for dihydroartemisinin (IC_50_; 0-6 hrs, 0.011 μM; 0-12 hrs, 0.009 μM) than that of chloroquine (IC_50_; 0-6 hrs, 0.15 μM; 0-12 hrs, 0.052 μM) in our previous study examining the activity of a different set of azithromycin analogues (Burns et al., 2020). As most antimalarials inhibit trophozoite or schizont stage parasites (Zhang et al., 1986; Cowman et al., 1988; Wilson et al., 2013), the fact these diverse analogues remained active with <12 hrs of treatment at concentrations similar to that required to kill parasites across 44 hrs is promising for azithromycin’s re-development as a quick-killing antimalarial.

To date, the mechanism of quick-killing for azithromycin and analogues has not been completely elucidated. Evidence from metabolomics studies using C1 from this study and analogues in a preceding study (Burns et al., 2020) indicate that some alterations in parasite metabolites with treatment of azithromycin and analogues resembled that observed for chloroquine, wherein a pattern of non-haemoglobin peptide is enriched (Creek et al., 2016). These data suggest that one of the sites of azithromycin analogues quick-killing activity could be the parasite’s food vacuole. Indeed, azithromycin is known to accumulate within acidic compartments of various cells (Kanoh and Rubin, 2010; Stepanic et al., 2011) and the cationic profile and lipophilic properties of azithromycin are improved through modification (Wilson et al., 2015; Peric et al., 2021) which may potentiate the accumulation and damage caused by the drug within acidic compartments. Further supporting this possibility, compound C1 also caused a build-up of peptides that could be linked back to haemoglobin, a signature not shared with azithromycin, dihydroartemisinin or chloroquine in these experiments that highlights that activity against the food vacuole may be an important mechanism for azithromycin analogues, in addition to other multi-factorial actions.

Interestingly, azithromycin and analogues do not completely mimic the activity of chloroquine. Firstly, azithromycin and a range of analogues tested with different modifications have activity against very early ring stage parasites, a stage of the lifecycle that chloroquine typically is considered to have poor activity against (Zhang et al., 1986; Wilson et al., 2013) (Burns et al., 2020), although a recent study suggests that faster growing *P. falciparum* lines can show sensitivity at early ring stages with 0-8 hour treatments (Murithi et al., 2020). The food vacuole itself is only fully formed by mid-ring stages (~12 hrs post invasion), removing the predominant site of chloroquine-like activity as a logical target during early ring stage development (Dluzewski et al., 2008; Abu Bakar et al., 2010). However, there is evidence that haemoglobin digestion occurs earlier in the lifecycle and therefore, azithromycin and analogues could act against this early digestive activity as the parasite develops (Abu Bakar et al., 2010; Xie et al., 2016). Secondly, the quick-killing activity of azithromycin and analogues is known to disrupt *Plasmodium* spp. merozoite invasion of RBCs as well as short term intracellular development and host-cell invasion of a related apicomplexan parasite, *Toxoplasma gondii* (Lee et al., 2011; Wilson et al., 2015). Neither *Plasmodium* merozoites nor *T. gondii* digest haemoglobin. Therefore, the quick-killing activity of azithromycin and analogues may encompass a number of mechanisms of action against the malaria parasite, but activity against the parasite’s food vacuole appears to be a target during trophozoite stages.

We found the lead analogues had minimal *in vitro* activity against the related apicomplexan parasite *T. gondii* and moderate activity against human cells, with most exhibiting >5-fold selectivity against *P. falciparum* parasites over human Huh-7D cells. This suggests that there may be limited general anti-Apicomplexa activity for these analogues, and that host toxicity warrants further evaluation if they are to be further developed as antimalarials. One caveat to this is that standard culture conditions, such as gas mix and growing medium, differ between malaria parasites and the *Toxoplasma* and mammalian cell cultures. Such differences have been shown to impact on activity of some drugs against malaria (Duffy and Avery, 2017) and it is a possibility that differences in standard tissue culture conditions could also impact on the activity of the drugs tested in this study.

An important consideration in developing azithromycin analogues as quick-killing antimalarials includes the safety and non-specific activity against the gut microbiome and other bacteria (Wei et al., 2018). The known mechanism of action of azithromycin against malaria parasites is the result of targeting of the bacterium-like ribosome of the parasite apicoplast (Sidhu et al., 2007). As an antimalarial, it would be highly desirable to develop azithromycin analogues that have both delayed death prophylaxis and quick-killing activities which could prevent parasite recrudescence, and reduce emergence of drug resistant parasites during treatment. However it is also possible that the remaining antibacterial activity could have off-target effects on the human microbiome and enrich for macrolide resistant pathogenic bacteria (Sutcliffe et al., 1996; Schroeder and Stephens, 2016; Wei et al., 2018). Thus, an alternative is to develop an azithromycin analogue featuring improved quick-killing for treatment of clinical malaria, but no activity against the bacterium-like ribosome. Azithromycin analogues tested in this study showed no significant activity against the apicoplast ribosome, with parasites lacking an apicoplast being equally susceptible to azithromycin analogues as wildtype parasites (Yeh and DeRisi, 2011; Uddin et al., 2018). Moreover, analogues lost potency relative to azithromycin against the azithromycin sensitive *S. pneumoniae* (strain D39). Together, these data suggest that modifications of the diverse azithromycin analogues tested in this study have largely removed activity against bacterium-like ribosomes, providing several new azithromycin-based structures to develop quick-killing specific antimalarials if desired.

## Conclusions

We have shown that azithromycin analogues with modest and variable efficacy against bacterial pathogens have improved quick-killing activity against blood-stage malaria parasites. The most potent analogues showed cross-species efficacy against both human and zoonotic *Plasmodium* species and 1.6 to 15-fold selectivity index for parasites over mammalian cells. Metabolomics analysis of drug treated, trophozoite stage parasites supports that the food vacuole of the parasite may be a site of drug activity, but the activity against early ring stage parasites indicates that they provide a broader spectrum of activity against blood stage parasite development than the clinically used food-vacuole targeting antimalarial chloroquine. Analogues showed limited efficacy against *T. gondii* and in assays that assessed activity against bacterium-like ribosomes, suggesting that quick-killing antimalarial activity can be selected over antibiotic activity for this diverse range of chemotypes. This study shows that these chemotypes may be appropriate starting points for development of antimalarials with broad blood stage quick-killing activity, but other azithromycin analogues would be preferred for the development of drugs that have favourable delayed-death and quick-killing dual mechanisms of action.

## Data availability statement

Metabolomics spectrometry data and search results have been deposited to the NIH Common Fund's National Metabolomics Data Repository (NMDR) website, the Metabolomics Workbench, https://www.metabolomicsworkbench.org where it has been assigned Project ID (ST001315). The data can be accessed directly via it's Project DOI: (10.21228/M8DT4D). Other datasets used in this study are available from the corresponding author on reasonable request.

## Author contributions

Study design and planning AB, BS, CG, DC, RO’H, JB, GM, DW. Performed experiments and generated reagents AB, MG, KM, GS, SF, DW. Provision of azithromycin analogues HV, SM. All authors contributed to the article and approved the submitted version.

## Funding

This work was made possible through the National Health and Medical Research Council of Australia (Project Grant 1143974 to DW, GM, BS, and CG; Development Grant 1113712 to BS; Career Development (II) Fellowship 1148700 to DC) and the Victorian State Government Operational Infrastructure Support and Australian Government NHMRC IRIISS. DW is a Hospital Research Foundation Fellow, BS is a Corin Centenary Fellow, JB is an NHMRC Investigator GrantFellow 1173046, AB was supported by an ARC-RTP scholarship. This work is supported by NIH grant U2C-DK119886.

## Acknowledgments

Human erythrocytes were kindly provided by the Red Cross Blood Bank (Adelaide, Australia). Metabolomics analysis was performed at the Monash Proteomics and Metabolomics Facility.

## Conflict of interest

The authors declare that the research was conducted in the absence of any commercial or financial relationships that could be construed as a potential conflict of interest.

## Publisher’s note

All claims expressed in this article are solely those of the authors and do not necessarily represent those of their affiliated organizations, or those of the publisher, the editors and the reviewers. Any product that may be evaluated in this article, or claim that may be made by its manufacturer, is not guaranteed or endorsed by the publisher.
