# Supplementary material for: Targeting malaria parasites with novel derivatives of azithromycin
Source: Front Cell Infect Microbiol. 2022 Nov 30;12:1063407. doi: 10.3389/fcimb.2022.1063407 (PMC9748569; doi:10.3389/fcimb.2022.1063407)
Supplement: Supplementary file 1 [file DataSheet_1.pdf]

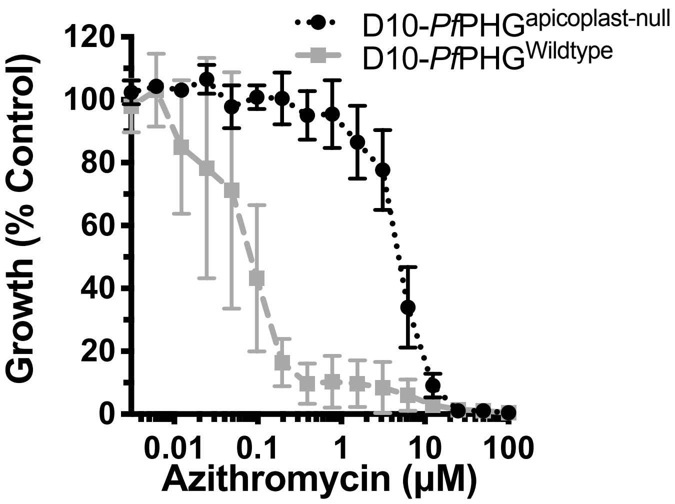

Supplementary Figure 1: Growth inhibition profiles of azithromycin in parasites lacking the apicoplast.

Early ring-stage *P. falciparum* parasites (0-4 hrs post-invasion) were treated with doubling dilutions of azithromycin and inhibition of growth measured for 2 cycle (delayed death, 120 hrs) assays (D10-PfPHG$^{apicoplast-null}$ IC_{50}, 4.5 μM; D10-PfPHG^{wildtype} IC_{50}, 0.07 μM. P= <0.0001). Parasitemia was measured at 120 hrs post invasion at schizont stage via flow cytometry. Data represents the means of 3 (or more) experiments expressed as percentage of non-inhibitory control and error bars represent ± SEM.

Supplementary Table 1: Activities of A-group azithromycin analogues

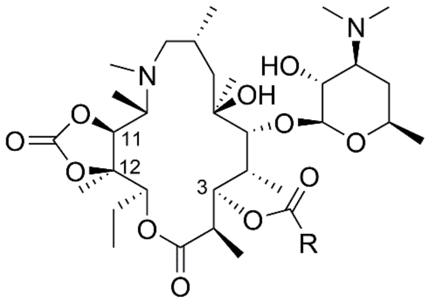

Compound ID	R-group	Carbamoyl substituent (R-group) name	Intracellular growth D10-PfPHG at 10 µM (% growth) [a]	Intracellular growth D10-PfPHG at 1 µM (% growth) [b]	Intracellular growth PkYH1 at 10 µM (% growth) [a]	Intracellular growth of PkYH1 at 1 µM (growth %) [b]	Original reference
A1	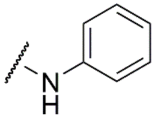	phenyl	93 (*6.8*)	92 (*2.4*)	79 (*11.9*)	95 (*2.4*)	5a (Yan et al., 2017)
A2	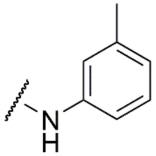	3-methylphenyl	3.3 (*3.2*)	16 (*7.9*)	3.3 (*1.1*)	16 (*7.7*)	5b (Yan et al., 2017)
A3	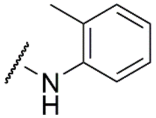	2-methylphenyl	3 (*2.6*)	6.8 (*4.1*)	1.9 (*0.3*)	11 (*7.9*)	5c (Yan et al., 2017)
A4	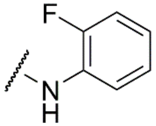	2-fluorophenyl	95 (*11.2*)	86 (*2.0*)	24 (*8.9*)	93 (*3.8*)	5d (Yan et al., 2017)

A5	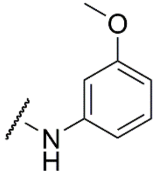	3-methyloxyphenyl	3.6 (*3.1*)	6.5 (*1.6*)	3.4 (*1.0*)	8.2 (*2.7*)	5e (Yan et al., 2017)
A6	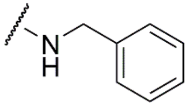	benzyl	93 (*8.3*)	94 (*3.7*)	70 (*9.2*)	97 (*2.6*)	5f (Yan et al., 2017)
A7	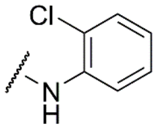	2-chlorophenyl	8.2 (*3.9*)	32 (*1.2*)	3.9 (*2.3*)	33 (*9.8*)	5g (Yan et al., 2017)
A8	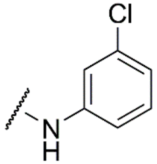	3-chlorophenyl	3.1 (*2.9*)	2.8 (*0.9*)	5.1 (*1.9*)	3.9 (*3.7*)	5h (Yan et al., 2017)
A9	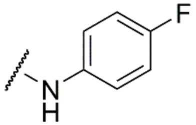	4-fluorophenyl	6.7 (*3.6*)	76 (*6.7*)	8.7 (*5.2*)	78 (*12.1*)	5i (Yan et al., 2017)
A10	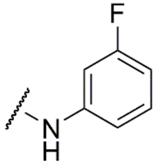	3-fluorophenyl	2.7 (*2.6*)	63 (*15*)	2.4 (*0.4*)	24 (*10.1*)	5j (Yan et al., 2017)
A11	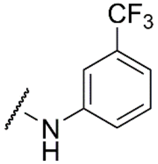	3-trifluorophenyl	11 (*9.3*)	90 (*5.3*)	24 (*5.7*)	21 (*10.6*)	5k (Yan et al., 2017)

A12	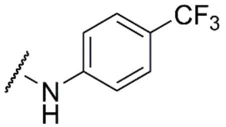	4-trifluorophenyl	2.7 (*2.7*)	13 (*8.2*)	5.8 (*3.0*)	0.9 (*0.5*)	5l (Yan et al., 2017)
A13	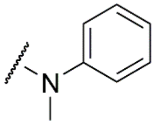	N-methyl-phenyl	3.4 (*2.8*)	12 (*9.9*)	1.1 (*0.4*)	1.8 (*1.6*)	5m (Yan et al., 2017)

[a] Drug treatment of intracellular growth, from rings to late schizonts, with no rupture cycle (D10-PfPHG, *P. falciparum,* 0-44 hrs or *Pk*YHI, *P. knowlesi* 0-28 hrs).

[b] Drug treatment of delayed death, from early-rings to late trophozoites, with two rupture cycle (D10-PfPHG, *P. falciparum,* 0-120 hrs or *Pk*YHI, *P. knowlesi* 0-78 hrs).

Data represents the means of 3 (or more) experiments expressed as percentage of non-inhibitory control.

Supplementary Table 2: Activities of B-group azithromycin analogues

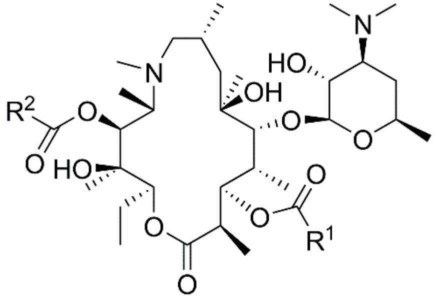

Compound ID	R-groups	Carbamoyl substituent (R-group) name	Intracellular growth D10-PfPHG at 10 µM (% growth) [a]	Intracellular growth D10-PfPHG at 1 µM (% growth) [b]	Intracellular growth PkYH1 at 10 µM (% growth) [a]	Intracellular growth of PkYH1 at 1 µM (growth %) [b]	Original reference
B1	R1 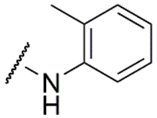	2-methylphenyl	2.3 (*2.2*)	18 (*15*)	4.3 (*2.0*)	2.5 (*1.5*)	6g (Yan et al., 2017)
	R2 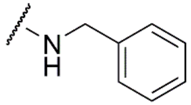	benzyl					
B2	R1 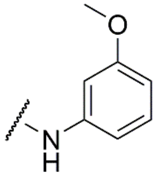	4-methyloxyphenyl	2.3 (*2.3*)	6.1 (*5.2*)	3.6 (*1.4*)	1.8 (*1.4*)	6h (Yan et al., 2017)

	R^2 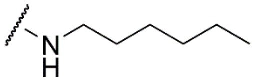	hexyl					

[a] Drug treatment of intracellular growth, from rings to late schizonts, with no rupture cycle (D10-PfPHG, *P. falciparum,* 0-44 hrs or *Pk*YHI, *P. knowlesi* 0-28 hrs).

[b] Drug treatment of delayed death, from early-rings to late trophozoites, with two rupture cycle (D10-PfPHG, *P. falciparum,* 0-120 hrs or *Pk*YHI, *P. knowlesi* 0-78 hrs).

Data represents the means of 3 (or more) experiments expressed as percentage of non-inhibitory control.

Supplementary Table 3: Activities of C-group azithromycin analogues

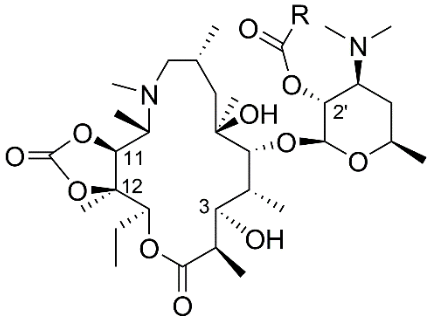

Compound ID	R-group	Carbamoyl substituent (R group) name	Intracellular growth D10-PfPHG at 10 µM (% growth) [a]	Intracellular growth D10-PfPHG at 1 µM (% growth) [b]	Intracellular growth PkYH1 at 10 µM (% growth) [a]	Intracellular growth of PkYH1 at 1 µM (growth %) [b]	Original reference
C1	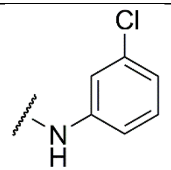	3-chlorophenyl	2.8 (*2.8*)	0.72 (*0.6*)	0.4 (*0.2*)	2.1 (*1.9*)	8e (Yan et al., 2017)
C2	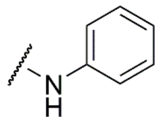	Phenyl	7.3 (*4.1*)	73 (*7.9*)	12 (*4.2*)	74 (*6.8*)	8a (Yan et al., 2017)

C3	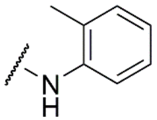	2-methylphenyl	5.6 (*5.5*)	67 (*9.8*)	1.6 (*0.5*)	73 (*13.4*)	8b (Yan et al., 2017)
C4	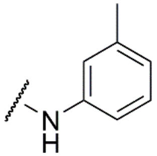	3-methylphenyl	85 (*15.8*)	89 (*7.6*)	74 (*9.6*)	96 (*3.2*)	8c (Yan et al., 2017)
C5	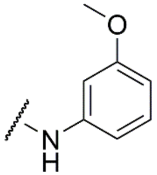	3-methyloxyphenyl	85 (*11.4*)	83 (*5.2*)	72 (*7.7*)	95 (*1.1*)	8d (Yan et al., 2017)

[a] Drug treatment of intracellular growth, from rings to late schizonts, with no rupture cycle (D10-PfPHG, *P. falciparum,* 0-44 hrs or *Pk*YHI, *P. knowlesi* 0-28 hrs).

[b] Drug treatment of delayed death, from early-rings to late trophozoites, with two rupture cycle (D10-PfPHG, *P. falciparum,* 0-120 hrs or *Pk*YHI, *P. knowlesi* 0-78 hrs).

Data represents the means of 3 (or more) experiments expressed as percentage of non-inhibitory control.

Supplementary Table 4: Activities of D-group azithromycin analogues

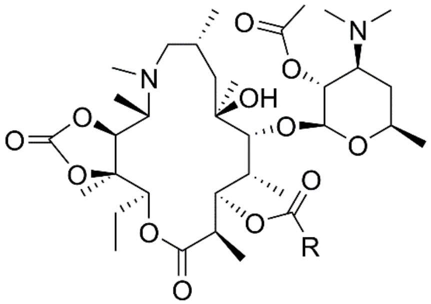

Compound ID	R-group	Carbamoyl substituent (R group) name	Intracellular growth D10-PfPHG at 10 µM (% growth) [a]	Intracellular growth D10-PfPHG at 1 µM (% growth) [b]	Intracellular growth PkYH1 at 10 µM (% growth) [a]	Intracellular growth of PkYH1 at 1 µM (growth %) [b]	Original reference
D1	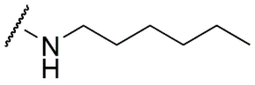	hexyl	3.2 (*2.5*)	2.5 (*1.4*)	1.2 (*0.3*)	85 (*8.3*)	(Yan et al., 2017)

D2	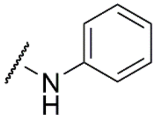	phenyl	31 (*16.3*)	77 (*7.7*)	12 (*5.7*)	84 (*5.9*)	(Yan et al., 2017)

[a] Drug treatment of intracellular growth, from rings to late schizonts, with no rupture cycle (D10-PfPHG, *P. falciparum,* 0-44 hrs or *Pk*YHI, *P. knowlesi* 0-28 hrs).

[b] Drug treatment of delayed death, from early-rings to late trophozoites, with two rupture cycle (D10-PfPHG, *P. falciparum,* 0-120 hrs or *Pk*YHI, *P. knowlesi* 0-78 hrs).

Data represents the means of 3 (or more) experiments expressed as percentage of non-inhibitory control

Supplementary Table 5: In cycle fold-change of analogues vs azithromycin

Compound	Intracellular growth D10-PfPHG IC_{50} (μM) [a]	Intracellular growth PkYH1 IC_{50} (μM) [a]	Fold difference analogue / Azithromycin D10-PfPHG	Fold difference analogue / Azithromycin PkYH1
Azithromycin	10.3 (*1.1*)	13 (*1.8*)		
A2	1.6 (*0.2*)	0.67 (*0.1*)	6.3	19
A3	1.5 (*0.07*)	1.1 (*0.3*)	6.7	12
A4	ND	7.8 (*1.0*)	ND	1.7
A5	1.8 (*0.3*)	1.7 (*0.5*)	5.6	7.6
A7	5.3 (*0.2*)	1.8 (*0.1*)	1.9	7.2
A8	1.4 (*0.2*)	0.81 (*0.2*)	7.1	16
A9	3.6 (*0.6*)	2.0 (*0.5*)	2.8	6.5
A10	4.2 (*0.5*)	1.5 (*0.3*)	2.4	8.7
A11	6.1 (*0.6*)	4.5 (*0.9*)	1.6	2.9
A12	1.8 (*0.8*)	1.3 (*0.08*)	5.6	10
A13	0.72 (*0.01*)	0.64 (*0.02*)	14	20
B1	1.6 (*0.4*)	1.3 (*0.2*)	6.3	10
B2	0.56 (*0.05*)	0.69 (*0.1*)	18	18.8
C1	0.3 (*0.02*)	1.4 (*0.2*)	30	9.2
C2	4.6 (*0.5*)	5.9 (*1.9*)	2.2	2.2
C3	3.2 (*0.2*)	3.9 (*0.6*)	3.1	3.3
D1	0.67 (*0.04*)	1.7 (*0.3*)	15	7.7
D2	3.7 (*0.3*)	2.9 (*0.4*)	2.7	4.5

[a] Drug treatment of intracellular growth, from rings to late schizonts, with no rupture cycle (D10-PfPHG, *P. falciparum,* 0-44 hrs or PkYHI, *P. knowlesi* 0-28 hrs). Data represents the means of 3 (or more) experiments expressed as percentage of non-inhibitory control.

ND= not done due to limited sample.

Supplementary Table 8: Delayed death fold-change of analogues vs azithromycin

Compound	120 hr growth D10-PfPHG IC_{50} (μM) [b] (±*SEM*)	120 hr growth *Pk*YH1 IC_{50} (μM) [b] (±*SEM*)	Fold difference analogue / Azithromycin D10-PfPHG	Fold difference analogue / Azithromycin PkYH1
Azithromycin	0.02 (*0.01*)	0.09 (*0.02*)		
A2	0.26 (*0.06*)	0.24 (*0.04*)	13	2.6
A3	0.47 (*0.09*)	0.36 (*0.05*)	13	4
A5	0.39 (*0.06*)	0.43 (*0.04*)	19	4.8
A7	0.76 *(0.02)*	0.54 *(0.08)*	38	6
A8	0.36 (*0.05*)	0.14 (*0.01*)	18	7
A9	0.82 (*0.08*)	0.69 (*0.08*)	41	7.7
A10	1.1 *(0.03)*	0.66 (*0.1*)	55	7.3
A11	ND	0.89 (*0.3*)	ND	9.9
A12	0.32 (*0.03*)	0.19 (*0.02*)	16	2.1
A13	0.18 (*0.02*)	0.39 (*0.03*)	9	4.3
B1	0.29 (*0.02*)	0.1 (*0.03*)	14	1.1
B2	0.14 (*0.01*)	0.09 (*0.03*)	7	1
C1	0.12 (*0.01*)	0.27 (*0.09*)	6	3
C2	1.1 *(0.1)*	ND	55	ND
C3	0.99 *(0.09)*	ND	50	ND
C4	2.8 *(0.4)*	ND	140	ND
C5	2.2 (*0.4*)	ND	110	ND
D1	0.12 *(0.03)*	0.75 *(0.4)*	6	8.3
D2	1.3 *(0.01)*	ND	65	ND

[b] Drug treatment of delayed death, from early-rings to late trophozoites, with two rupture cycles (D10-PfPHG, *P. falciparum,* 0-120 hrs or PkYHI, *P. knowlesi* 0-78 hrs).

Data represents the means of 2 (or more) experiments expressed as percentage of non-inhibitory control.

ND= not done due to limited sample.

Supplementary Table 9: Activity of analogues against bacterial ribosomes

Compound	MIC average [a] (±*SEM*)	44 hr growth D10-PfPHG$^{Apicoplast-null}$ IC_{90} (% growth) [b] (±*SEM*)	120 hr growth D10-PfPHG$^{Apicoplast-null}$ IC_{90} (% growth) [c] (±*SEM*)
Azithromycin	0.125 *(0.0)*	0.6 *(0.1)*	95 *(3.9)*
A1	>16 *(0.0)*	ND	ND
A2	4 *(0.0)*	1.0 *(0.7)*	13 *(1.1)*
A3	2 *(0.0)*	6.9 *(2.6)*	10 *(1.9)*
A4	4 *(0.0)*	ND	ND
A5	8 *(0.0)*	6.4 *(1.2)*	12 *(1.3)*
A6	>16 *(0.0)*	ND	ND
A7	4 *(0.0)*	1.1 *(0.4)*	ND
A8	2 *(0.0)*	1.4 *(0.4)*	ND
A9	4 *(0.6)*	5.9 *(1.7)*	9.8 *(1.2)*
A10	8 *(0.0)*	0.05 *(0.05)*	7.3 *(5.2)*
A11	>16 *(0.0)*	0.12 *(0.05)*	ND
A12	4 *(1.3)*	0.62 *(0.4)*	9.6 *(5.3)*
A13	1 *(0.0)*	4.8 *(3.0)*	17 *(3.4)*
B1	0.25 *(0.0)*	3.2 *(1.6)*	6.9 *(2.9)*
B2	8 *(2.6)*	10 *(4.4)*	15 *(7.0)*
C1	16 *(2.6)*	11 *(5.8)*	23 *(4.9)*
C2	4 *(0.0)*	5.8 *(3.1)*	12 *(1.4)*
C3	8 *(1.3)*	9.6 *(3.3)*	11 *(2.6)*
C4	>16 *(0.0)*	ND	6.4 *(2.1)*
C5	8 *(0.0)*	8.9 *(2.4)*	15 *(4.5)*
D1	8 *(1.3)*	13 *(2.7)*	8.1 (3.8)
D2	8 *(1.3)*	9.9 *(3.5)*	10.3 *(1.4)*

[a] Minimum inhibitory concentration (MIC) were determined as described in Methods. MIC dilution series were analysed individually, and values are represented as (µM). The MIC data represents the means of 3 or more experiments expressed as a percentage of non-inhibitory control.

[b] Drug treatment of D10-PfPHG$^{apicoplast-null}$ parasites supplemented with IPP for intracellular growth, from rings to late schizonts, with no rupture cycle (*P. falciparum,* 0-44 hrs).

[c] Drug treatment of D10-PfPHG$^{apicoplast-null}$ supplemented with IPP for delayed death assays, from early-rings to late trophozoites, with two rupture cycle (*P. falciparum,* 0-120 hrs).

Data represents the means of 3 (or more) experiments expressed as percentage of non-inhibitory control.

ND= not done due to limited sample.
