# Supplementary material for: Targeting malaria parasites with novel derivatives of azithromycin
Source: Front Cell Infect Microbiol. 2022 Nov 30;12:1063407. doi: 10.3389/fcimb.2022.1063407 (PMC9748569; doi:10.3389/fcimb.2022.1063407)
Supplement: Supplementary file 2 [file DataSheet_2.pdf]

Supplementary Table 6 List of peptides dysregulated that do not map to haemoglobin α (HBA) and β (HBB) subunits following treatment with azithromycin analogue C1, azithromycin (AZ), chloroquine (CQ), and DHA. The heatmap is expressed as the mean fold change versus ethanol control from three technical replicates within two independent experiments (Experiment 1 and Experiment 2). Yellow indicates no change, white indicates not detected, while red and blue indicates increased and decreased abundances respectively. Bold denotes changes that were statistically significantly different, $p<0.05$. Order of amino acids listed has not been confirmed using MS/MS.

	Experiment 1				Experiment 2			
	AZR	CQ	DHA	C1	AZR	CQ	DHA	C1
**Val-Gly**	**1.29**	**0.62**	**0.33**	**2.66**	**1.04**	**1.10**	**1.24**	**2.50**
**Pro-Ser**	**3.75**	**2.15**	**0.62**	**5.14**	**6.44**	**9.37**	**0.91**	**7.01**
**Leu-Pro**	**0.90**	**1.43**	**0.71**	**2.27**	**0.19**	**4.35**	**0.74**	**4.47**
**Ala-Ala-Ala**				**10.55**	**79.13**	**143.45**		**137.02**
**Ile-Thr**	**5.85**	**1.73**	**0.11**	**3.22**	**5.75**	**4.57**	**0.50**	**3.17**
**Lys-Ser**	**2.90**	**1.72**	**0.85**	**2.55**	**1.97**	**3.66**	**0.99**	**3.12**
**Met-Pro**	**29.01**	**16.74**	**0.97**	**20.50**	**168.34**	**294.61**		**170.79**
**Pro-His**	**1.75**	**0.70**	**0.35**	**4.50**	**0.92**	**0.89**	**0.90**	**3.27**
**Lys-Asn**	**33.95**	**23.15**		**17.58**	**74.45**	**86.06**	**2.65**	**55.58**
**Lys-Asp**	**0.82**	**0.51**	**0.69**	**5.85**	**0.16**	**0.30**	**0.88**	**2.04**
**Ser-Arg**	**19.03**	**11.29**	**0.86**	**5.28**	**13.50**	**26.69**		**11.56**
**Asn-His**	**3.15**	**1.34**	**0.62**	**2.72**	**3.90**	**6.35**	**1.19**	**3.12**
**Pro-Arg**	**1.30**	**1.42**	**0.82**	**2.72**	**2.40**	**4.21**	**0.84**	**4.15**
**Pro-Tyr**	**113.67**	**35.77**		**60.72**	**68.16**	**117.98**		**50.56**
**Asn-Arg**	**13.72**	**8.31**	**1.31**	**7.13**	**5.72**	**5.10**	**0.37**	**3.00**
**Ala-Val-Gly-Pro**	**0.58**	**0.54**	**0.55**	**2.38**	**0.07**	**0.76**	**1.23**	**2.24**
**Lys-Trp**	**57.28**	**76.64**		**53.54**	**104.49**	**383.45**		**74.95**
**Glu-Pro-Glu**	**1.69**	**0.99**	**0.65**	**7.76**	**0.46**	**1.03**	**0.89**	**3.38**

Supplementary Table 7 List of peptides dysregulated that map to haemoglobin α (HBA) and β (HBB) subunits following treatment with azithromycin analogue C1, azithromycin (AZ), choloroquine (CQ), and DHA. The heatmap is expressed as the mean fold change versus ethanol control from three technical replicates within two independent experiments (Experiment 1 and Experiment 2). Yellow indicates no change, white indicates not detected, while red and blue indicates increased and decreased abundances respectively. Not underlined peptides were mapped to HBA, while underlined peptide were mapped to HBB. Bold denotes changes that were statistically significantly different, $p<0.05$.

	Experiment 1				Experiment 2			
	AZR	CQ	DHA	C1	AZR	CQ	DHA	C1
**Pro-Ala**	**0.58**	**0.51**	**0.43**	**2.64**	0.44	0.64	0.90	**2.74**
**Pro-Ala-Asp-Lys**	2.20	0.86	**0.47**	**9.09**	1.46	1.00	0.64	**5.03**
**Pro-Asn**	**1.23**	0.81	**0.46**	**3.77**	1.26	**1.57**	0.86	**3.68**
**Val-Gly-Ala**	1.49	0.85	0.15	**2.85**	1.08	1.90	0.90	**2.91**
**Arg-Val**	**9.81**	**5.66**	0.92	**6.92**	**6.30**	**9.50**	0.53	**4.93**
**Leu-Arg**	**2.75**	1.40	**0.28**	**2.29**	**4.34**	**6.82**	0.66	**3.17**
**Pro-Ala-Asp**	0.85	0.69	**0.60**	**6.96**		**0.53**	0.82	**2.53**
**Pro-Ala-Glu**	0.59	**0.52**	**0.57**	**4.11**		**0.22**	1.16	**2.07**
**Pro-Val-Asn**				**24.74**			**6.28**	**219.41**
**Lys-Gly**	0.52	0.14	**0.29**	**3.36**	**0.38**	0.71	1.21	**2.40**
**Glu-Ala**	0.75	0.51	0.54	**2.35**	**1.77**	1.70	**0.75**	**4.69**
**Pro-Glu**	0.96	0.74	**0.53**	**4.11**	0.56	0.79	0.91	**4.07**
**Thr-Gln**	**1.95**	1.28	0.09	**2.81**	**6.48**	7.99	1.03	**7.00**
**Glu-Lys**	**8.74**	**3.83**	0.69	**7.14**	**5.91**	**10.98**	0.20	**5.76**
**Pro-Glu-Asn**	**7.88**	1.50		**112.06**	**13.75**			**255.86**
**Val-Asp-Glu**		1.18		**30.30**		**3.29**		**41.85**
**Pro-Trp-Thr**		0.66	0.54	**3.70**		0.81	0.78	**3.00**
**Pro-Pro-Val-Gln**	**1.90**	0.64	**0.37**	**6.63**	0.81	0.88	0.80	**3.52**
**Pro-Gln-Gln-Lys**	**3.26**	0.61	0.17	**5.04**	**2.08**	1.06	0.79	**4.30**
**Pro-Trp-Thr-Gln**	0.75	0.97	0.15	**11.00**		1.24	0.59	**5.13**
